# Aldosterone Blockade in Acute Myocardial Infarction: A Systematic Review and Meta-Analysis

**DOI:** 10.1155/2021/1710731

**Published:** 2021-10-25

**Authors:** Qiao Chen, Die Zhao, Jie Sun, Chengzhi Lu

**Affiliations:** ^1^The First Central Clinical School, Tianjin Medical University, No. 22 Qixiangtai Road, Heping District, Tianjin 300070, China; ^2^Department of Medical Psychology, The Basic Medicine College of Tianjin Medical University, No. 22 Qixiangtai Road, Heping District, Tianjin 300070, China; ^3^Department of Cardiology, Tianjin First Central Hospital, No.24 Fukang Road, Nankai District, Tianjin 300192, China

## Abstract

**Background:**

A comprehensive evaluation of the benefits of mineralocorticoid receptor antagonists (MRA) in acute myocardial infarction (AMI) patients is lacking.

**Objective:**

To summarize the evidence on the efficacy and safety of MRA in patients admitted for AMI.

**Methods:**

Articles were identified through PubMed, Embase, Cochrane Library, Ovid (Medline1946-2021), and ClinicalTrials.gov databases from their inception to December 31, 2020.

**Results:**

15 articles with a total of 11,861 patients were included. MRA reduced the risk of all-cause mortality by 16% (relative ratio (RR): 0.84; 95% confidence interval (CI) (0.76, 0.94); *P* = 0.002) and the incidence of cardiovascular adverse events by 12% (RR: 0.88, 95% CI (0.83, 0.93), *P* < 0.00001) in post-AMI patients, and further analysis demonstrated that early administration of MRA within 7 days after AMI resulted in a greater reduction in all-cause mortality (RR: 0.72, 95% CI (0.61, 0.85), *P* < 0.0001). Subgroup analyses showed that post-STEMI patients without left ventricular systolic dysfunction (LVSD) treated with MRA had a 36% reduction in all-cause mortality (RR: 0.64, 95% CI (0.46, 0.89), *P* = 0.007) and a 22% reduction in cardiovascular adverse events (RR: 0.78, 95% CI (0.67, 0.91), *P* = 0.002). Meanwhile, post-STEMI patients without LVSD treated with MRA get significant improvements in left ventricular ejection fraction (mean difference (MD): 2.69, 95% CI (2.44, 2.93), *P* < 0.00001), left ventricular end-systolic index (MD: -4.52 ml/m^2^, 95% CI (-8.21, -0.83), *P* = 0.02), and left ventricular end-diastolic diameter (MD: -0.11 cm, 95% CI (-0.22, 0.00), *P* = 0.05). The corresponding RR were 1.72 (95% CI (1.43, 2.07), *P* < 0.00001) for considered common adverse events (hyperkalemia, gynecomastia, and renal dysfunction).

**Conclusions:**

Our findings suggest that MRA treatment reduces all-cause mortality and cardiovascular adverse events in post-AMI patients, which is more significant in patients after STEMI without LVSD. In addition, MRA treatment may exert beneficial effects on the reversal of cardiac remodeling in patients after STEMI without LVSD.

## 1. Introduction

Aldosterone, a major mineralocorticoid receptor agonist, is primarily synthesized in the adrenal cortex [1]. Elevated aldosterone levels after AMI have been shown to correlate with deterioration of cardiac function and worse adverse clinical outcomes [2–5] through promoting the development and progression of ventricular remodeling [6, 7]. Globally, despite remarkable advances in the prevention, diagnosis, and treatment [8], AMI has been a serious threat to human health [9], with an increase in young patients, especially in developed countries [10]. Since the EPHESUS trial in 2003 [11] (Eplerenone Post-Acute Myocardial Infarction Heart Failure Efficacy and Survival Study) established morbidity and mortality benefits of aldosterone blockade with eplerenone in post-AMI patients, MRA have been used to treat patients admitted for AMI for nearly two decades. However, in 2016, the ALBATROSS [12] (Aldosterone Lethal effects Blockade in Acute myocardial infarction Treated with or without Reperfusion to improve Outcome and Survival at Six months follow-up, NCT01059136) trial and then the current MINIMIZE STEMI [13] (Mineralocorticoid receptor antagonist pretreatment to MINIMISE reperfusion injury after ST-elevation myocardial infarction, NCT01882179) trial have shown little cardiovascular benefits, raising the question of whether AMI subtypes, treatment initiation time, and duration, or left ventricular ejection fraction (LVEF) affect the clinical efficacy of MRA. Given the cumulative data on this topic, a comprehensive evaluation is required to provide favorable support.

## 2. Materials and Methods

This meta-analysis was performed and reported according to the recommendations of the Cochrane Collaboration [14] and the preferred reporting items for systematic reviews and meta-analyses (PRISMA) guidelines [15] (Supplementary material 1). The protocol of the present meta-analysis was registered under PROSPERO (https://www.crd.york.ac.uk/prospero/display_record.php?ID=CRD42021230790).

### 2.1. Search Strategy

Articles were searched through electronic databases. Details of full search strategy are provided in Supplemental material 2. The inclusion criteria were as follows: (1) included post-AMI patients; (2) clinical prospective randomized controlled trials (RCTs), with groups divided into MRA and non-MRA; (3) compared with standard therapy or placebo or both; (4) having a study duration ≥ 4 weeks and a sample size ≥ 40 patients; (5) used the drugs of interest (spironolactone, eplerenone, and canrenoate); (6) reported at least one of the outcomes of interest; and (7) published in English. The search was supplemented by reviewing reference lists and hand-searching relevant journals for further potential studies.

### 2.2. Trial Selection

Two investigators (Qiao Chen and Die Zhao) independently obtained eligible articles. Discrepancies were discussed with a third reviewer (Jie Sun) until consensus was reached. If necessary, we contacted the original authors to avoid involving the same or partially identical subjects recruited in ≥ 1 trial by the same group.

### 2.3. Data Extraction and Synthesis

A standardized data collection form was used to systematically extract information from each report, including study and patient characteristics (Table 1 and Table 2), data on changes in cardiac structure and function from baseline to follow-up, numbers of major clinical outcomes, and adverse events. We used definitions of hyperkalemia, renal dysfunction, and gynecomastia based on primary publications. Hypokalemia was defined as a potassium level < 3.5 mmol/L. LSVD was determined by LVEF ≤ 40%. If a given trial could be divided into ≥ 2 separate studies due to different treatment time points, we extracted data from the most recent or most complete publications. Different dose groups in the same study were independently included. We extracted the number of populations with different treatment initiation times from a substudy of the EPHESUS trial [16].

### 2.4. Quality Assessment

We used the Cochrane Collaboration risk of bias tool and the Modified Jadad scoring system [29, 30] to assess the overall quality of included studies. Modified Jadad scores were calculated by assessing adequate randomization, allocation concealment, double-blinding, and withdrawals and dropouts per treatment group. Score ≤ 4 was defined as low-quality reports.

### 2.5. Statistical Analysis

Meta-analysis was performed by Review Manager version 5.3 and Stata version 16.0. Heterogeneity was assessed by Cochran's *Q* test, and *P* < 0.10 was considered significant [31]. The inconsistency index (*I*^2^) was used to estimate the level of heterogeneity among studies. 25%, 50%, and 75% corresponded to low, medium, and high levels. Data were pooled using a fixed effects model, when *I*^2^ values were below 50%; otherwise, a random effects model was used. If similar estimates were obtained by both methods, we only reported the random effects results to cover possible heterogeneity, because three drugs and different patients were included particularly in control groups. Data were presented as RR or MD with 95% CI. Two-tailed *P* < 0.05 was considered statistically significant. Subgroup analyses were conducted according to LVEF, treatment initiation time and duration, and AMI subtypes. Sensitivity analyses were carried out by sequentially excluding each trial one from the total studies at a time and recalculating the difference estimates for remaining trials. Publication bias was assessed with funnel plots and the Egger's test, and *P* < 0.10 was considered statistically significant.

## 3. Results

### 3.1. Study Characteristics

**Figure 1:**
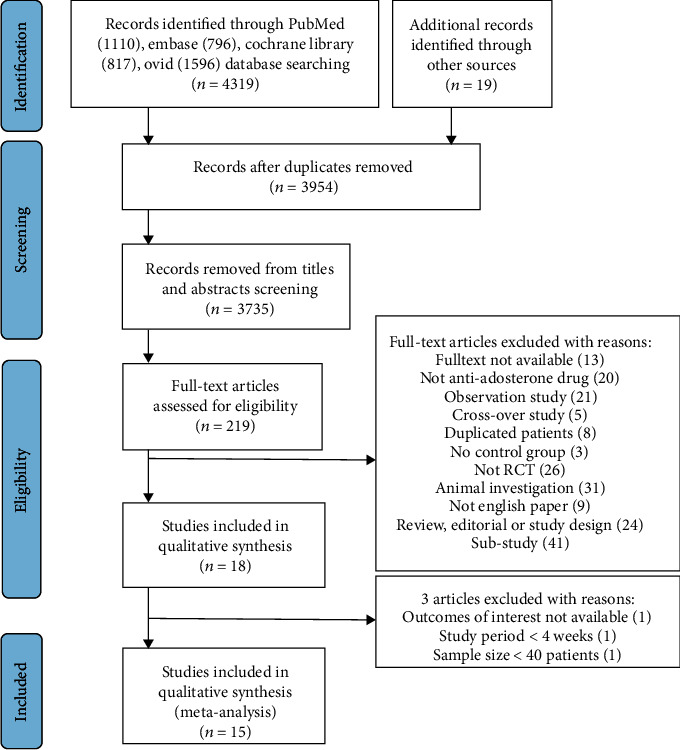
Preferred reporting items for systematic reviews and meta-analyses flow diagram. This flowchart records the process of literature screening and the reasons for exclusion.

**Figure 2:**
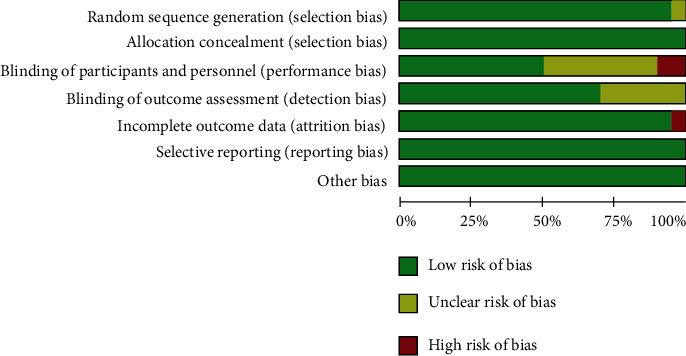
The risk of bias graph of the included studies. Green represents low risk, yellow represents unclear risk, and red represents high risk.

We found 4338 potentially articles, among which 15 trials [11–13, 17–28] involving 11,861 individuals were included (Figure 1). Treatment duration ranged from 1 to 24 months (8.40 ± 5.77). Patients were randomized to receive spironolactone in 8 trials (*n* = 1462), eplerenone in 4 trials (*n* = 4081), and canrenoate in 3 trials (*n* = 459) and assigned 1408, 3990, and 461 patients to control groups, respectively. The EPHESUS trial [11] accounted for more than half of the patients. Two studies [12, 25] did not use double-blind methods, and one study [24] reported incomplete outcome data (Figure 2). The kappa statistic 0.83 (95% CI: 0.52 to 1.14) showed a good agreement between two reviewers (Supplemental material 3). The Modified Jadad scores of trials varied from 5 to 7 points, indicating that this meta-analysis was a relatively high-quality report.

### 3.2. All-Cause Mortality

**Figure 3:**
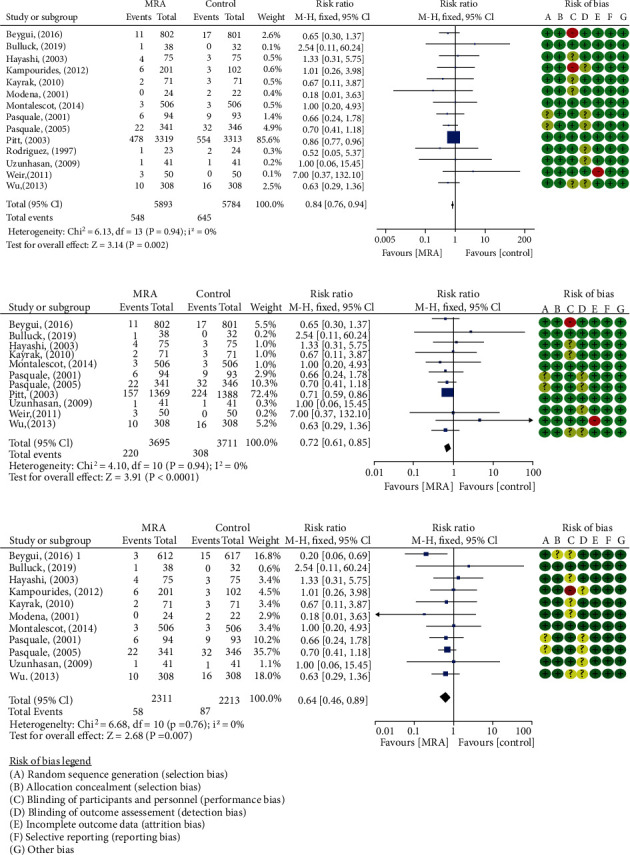
Forest plots of meta-analysis for comparison of all-cause mortality between the two groups: (a) all-cause mortality of post-AMI patients; (b) all-cause mortality of patients administrated MRA within 7 days after AMI; (c) all-cause mortality of post-STEMI patients without LVSD.

**Figure 4:**
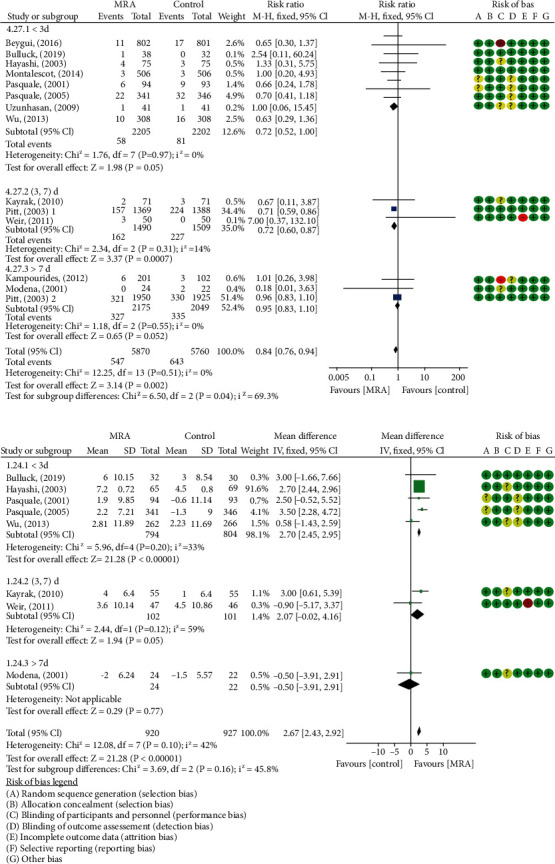
Forest plots of meta-analysis for subgroup analysis based on MRA treatment initiation time between the two groups: (a) all-cause mortality; (b) LVEF.

**Figure 5:**
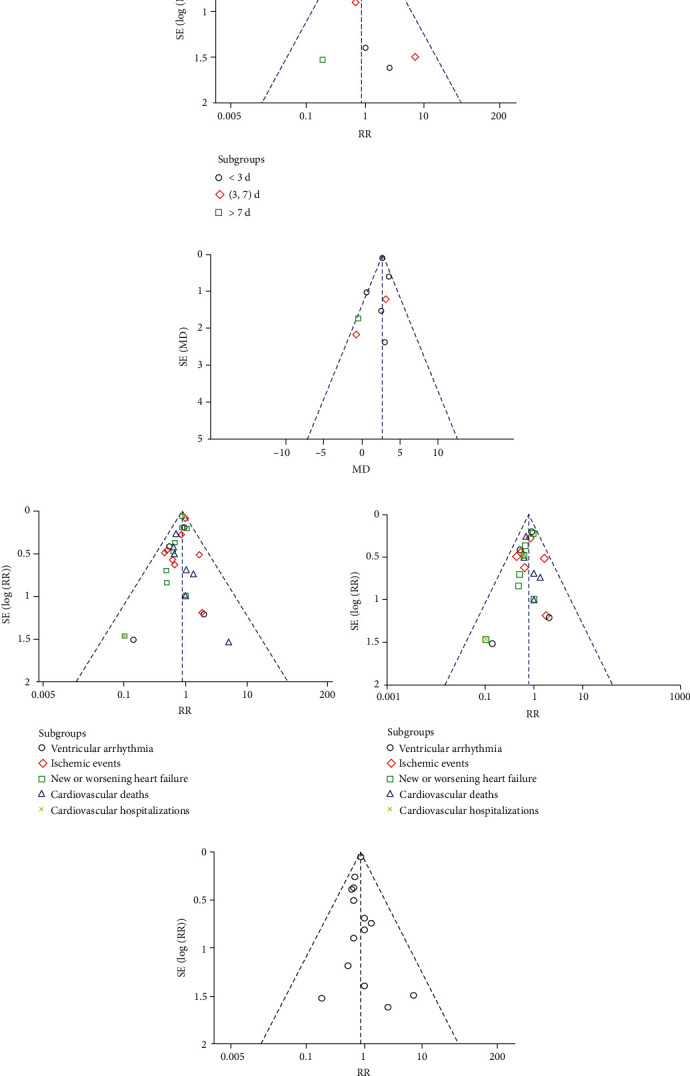
Funnel plots depicting the publication bias: (a) all-cause mortality based on treatment initiation time; (b) LVEF based on treatment initiation time; (c) cardiovascular adverse events in post-AMI patients; (d) cardiovascular adverse events in post-STEMI patients without LVSD; (e) all-cause mortality of post-AMI patients.

14 studies [11–13, 17–26, 28] including 11,677 post-AMI patients reported all-cause mortality. 548/5893 (9.30%) and 645/5784 (11.15%) were observed in MRA and control arms, respectively, with a general reduction of 16% (RR: 0.84, 95% CI (0.76, 0.94), *P* = 0.002, *I*^2^ = 0%, Figure 3). The reduction benefit was particularly evident in post-STEMI patients without LVSD (RR: 0.64, 95% CI (0.46, 0.89), *P* = 0.007, *I*^2^ = 0%, Figure 3). Early administration of MRA within 7 days resulted in a significant reduction in death after randomization (RR: 0.72, 95% CI (0.61, 0.85), *P* < 0.0001, *I*^2^ = 0%, Figure 3). In addition, further subgroup analyses showed a 28% reduction in all-cause mortality of post-AMI patients who initiated MRA treatment within 3 days or (3, 7) days (RR: 0.72, 95% CI (0.52, 1.00), *P* = 0.05, *I*^2^ = 0%; RR: 0.72, 95% CI (0.60, 0.87), *P* = 0.0007, *I*^2^ = 14%, Figure 4). No evidence of publication bias as suggested by funnel plot and the Egger's test (*P* = 0.41) was observed (Figure 5). None of the individual studies significantly influenced the pooled all-cause mortality estimates in the leave-one-out sensitivity.

### 3.3. New or Worsening HF and Deaths due to HF

**Figure 6:**
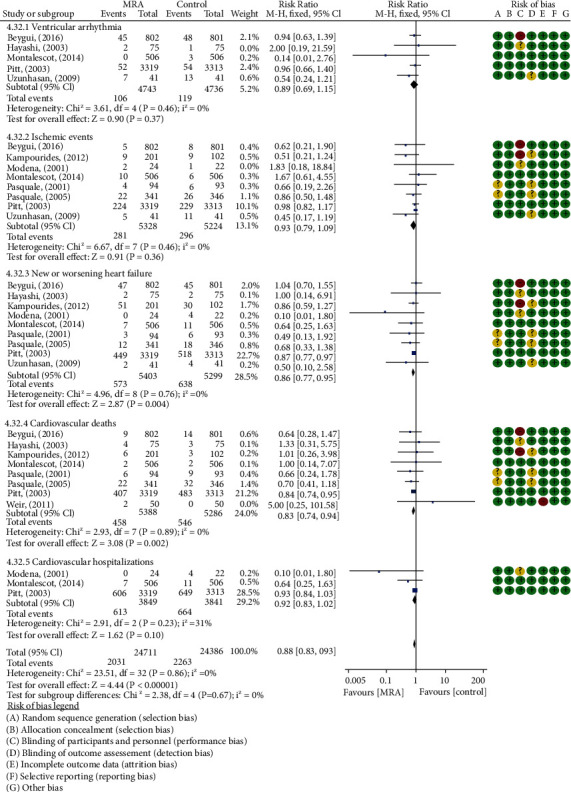
Forest plots of meta-analysis for comparison of cardiovascular adverse events in post-AMI patients between the two groups.

Nine RCTs [11, 12, 18–22, 25, 28] involving 10,702 post-AMI patients showed a significant 14% reduction (10.61% in the MRA groups vs. 12.04% in the control groups) in new or worsening heart failure (HF) after MRA treatment (Figure 6). Excluding the EPHESUS trial [11] with a weight of 79.9%, RR resulted in no statistical significance: from (0.86, 95% CI (0.77, 0.95), *P* =0.004, *I*^2^ = 0%) to (0.83, 95% CI (0.65, 1.05), *P* =0.12, *I*^2^ = 0%). Deaths due to HF were reported in above all but four trials [11, 12, 19–21] with overall 9259 post-AMI patients. Overall, MRA treatment was associated with a reduced risk of deaths due to HF (Table 3); a weight of 78.5% came from the EPHESUS trial [11]. None of the individual studies influenced the pooled estimate of deaths due to HF.

### 3.4. Composite Outcomes of Cardiovascular Adverse Events

**Figure 7:**
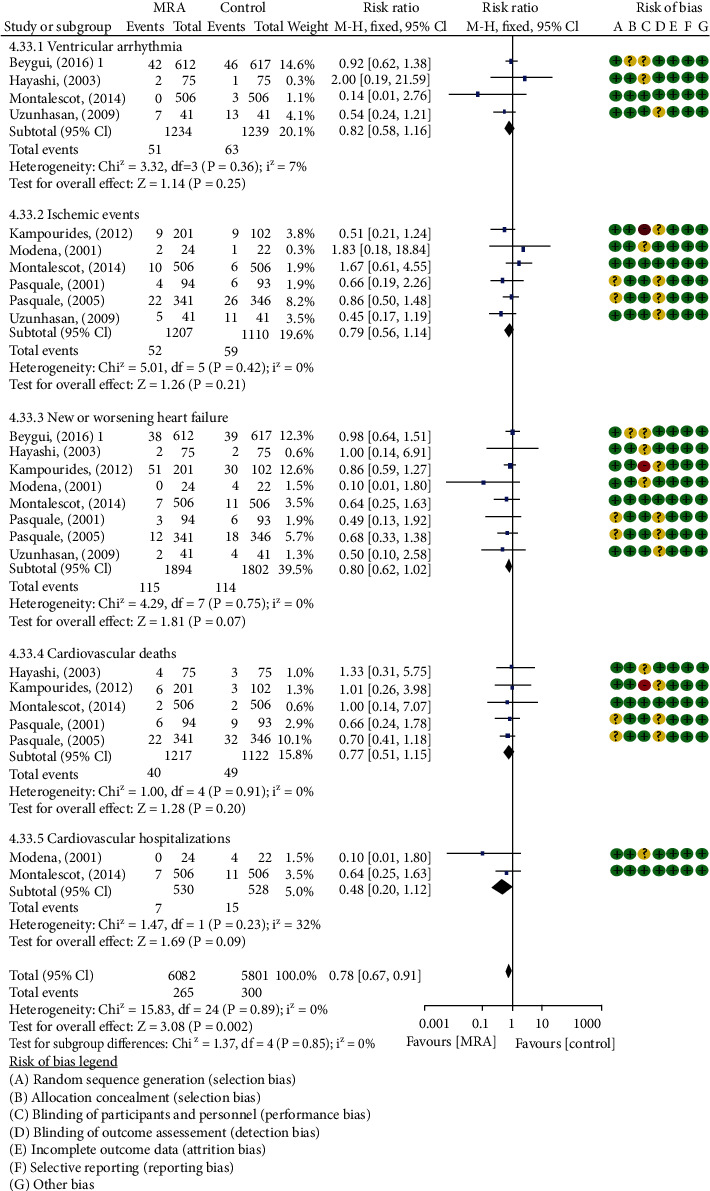
Forest plots of meta-analysis for comparison of cardiovascular adverse events in post-STEMI patients without LVSD between the two groups.

Ten studies [11, 12, 18–22, 24, 25, 28] involving 10,802 post-AMI patients (5453 in the MRA groups vs. 5349 in the control groups) reported the composite outcomes for ventricular arrhythmia, ischemic events, new or worsening HF, cardiovascular deaths, and cardiovascular hospitalizations. Overall, 8.22% of control and 9.28% of aldosterone-blockade patients reported 4294 cardiovascular adverse events (ventricular arrhythmia: 106/119; ischemic events: 281/296; new or worsening HF: 573/638; cardiovascular deaths: 458/546; cardiovascular hospitalizations: 613/664) over a median follow-up of 9.30 months. MRA were associated with a 12% reduction in the risk of cardiovascular adverse events (RR: 0.88, 95% CI (0.83, 0.93), *P* < 0.00001, *I*^2^ = 0%, Figure 6). In addition, 8 studies [12, 18–22, 24, 25, 28] involving 3696 post-STEMI patients without LVSD reported 565 cardiovascular adverse events (ventricular arrhythmia: 51/63; ischemic events: 52/59; new or worsening HF: 115/114; cardiovascular deaths: 40/49; cardiovascular hospitalizations: 7/15) over a median follow-up of 8.94 months. Cardiovascular adverse events were observed in 4.36% of patients in the MRA groups versus 5.17% in the control groups. Subgroup analyses showed reduction benefits were particularly evident in post-STEMI patients without LVSD; MRA were associated with a 22% reduction in the risk of cardiovascular adverse events (RR: 0.78, 95% CI (0.67, 0.91), *P* = 0.002, *I*^2^ = 0%, Figure 7). No evidence of publication bias was found for each outcome at visual inspection of funnel plots or Egger's test (all *P* > 0.10) (Figure 5).

### 3.5. Changes of Cardiac Structure and Function

**Figure 8:**
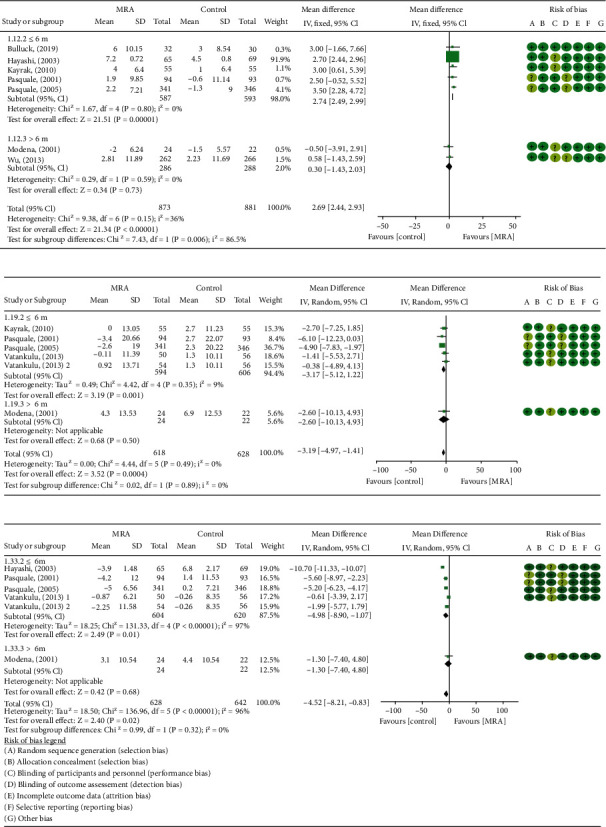
Forest plots of meta-analysis for comparison of cardiac ultrasound parameters based on treatment duration in post-STEMI patients without LVSD between the two groups: (a) LVEF; (b) LVEDVI; (c) LVESVI.

The effects of MRA on changes in LVEF of post-AMI patients were investigated in 8 studies [13, 18–21, 23, 24, 26] that included 920 patients treated with MRA and 927 patients treated without MRA. The analysis of the overall effects showed a significant difference in changes in LVEF (MD: 2.33, 95% CI (1.47, 3.19), *P* < 0.00001, Figure 4) between post-AMI patients who were treated with or without MRA with low heterogeneous results (*I*^2^ = 42%). Subgroup analyses showed a 2.64% improvement in LVEF in post-AMI patients who initiated MRA treatment within 3 days (MD: 2.64, 95% CI (1.88, 3.40), *P* < 0.00001, *I*^2^ = 33%, Figure 4). In addition, for post-STEMI patients without LVSD under MRA treatment, improvement in LVEF, LVEDVI, and LVESVI was apparent, and further analysis demonstrated a reduction in LVEDD but not in LVESD (Table 3). Further subgroup analyses were undertaken for LVEF, LVESVI, and LVEDVI by treatment durations. As expected, in post-STEMI patients without LVSD, significance was found in trials followed ≤6 months; as the durations increased, the extent of reduction in LVEF, LVESVI, and LVEDVI was weakened or became nonsignificant (LVEF—MD: 2.74, 95% CI (2.49, 2.99), *P* < 0.00001, *I*^2^ = 0%; LVESVI—MD: −4.98 ml/m^2^, 95% CI (−8.90, −1.07), *P* = 0.01, *I*^2^ = 97%; LVEDVI—MD: −3.17 ml/m^2^, 95% CI (−5.12, −1.22), *P* = 0.001, *I*^2^ = 9%, Figure 8). Except LVEF (*P* = 0.006, *I*^2^ = 86.5%), no significant differences were observed between subgroups (LVESVI: *P* = 0.32, *I*^2^ = 0%; LVEDVI: *P* = 0.89, *I*^2^ = 0%). The E/A ratio and EDT were reported in 4 studies [20, 21, 23, 27] with 1200 post-STEMI patients without LVSD. The results of meta-analysis showed that MRA significantly increased E/A ratio and prolonged EDT (Table 3).

### 3.6. Adverse Reactions

**Figure 9:**
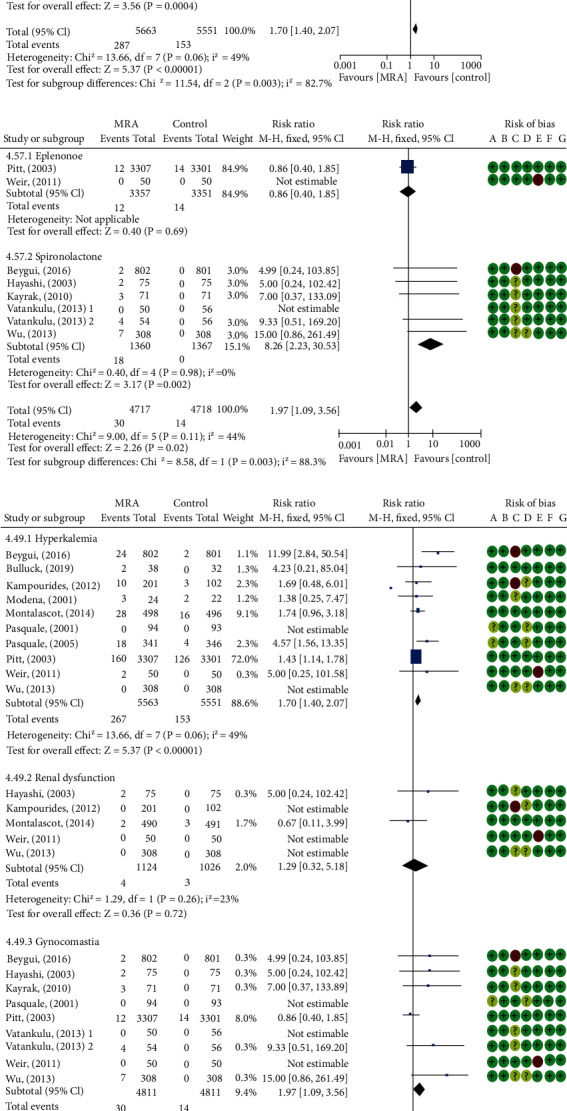
Forest plots of meta-analysis for comparison of adverse events between the two groups: (a) subgroup analysis of hyperkalemia based on drug types; (b) subgroup analysis of gynecomastia based on drug types; (c) all considered adverse events.

Hyperkalemia, renal dysfunction, and gynecomastia were the main observed side effects of MRA in the 15 included studies. MRA increased serum potassium and creatinine levels (Table 3), a corresponding increase in the incidence of renal dysfunction was found, but this result lacked statistical significant (RR: 1.29, 95% CI (0.32, 5.18), *P* = 0.72, *I*^2^ = 23%, Figure 9). A higher rate of hyperkalemia was 4.71% in the MRA arms versus 2.77% in control groups. In contrast, hypokalemia occurred less frequently in MRA groups (Table 3). Gynecomastia occurred in experiment (0.62%) and control (0.29%) patients. In general, the incidence of all considered adverse events nearly doubled in patients treated with MRA, compared to those receiving placebo or standard therapy (RR: 1.72, 95% CI (1.43, 2.07), *P* < 0.00001, *I*^2^ = 37%, Figure 9). Subgroup analyses showed spironolactone significantly increased the risk of hyperkalemia and gynecomastia (RR: 10.33, 95% CI (2.85, 37.41), *P* = 0.0004, *I*^2^ = 0%; RR: 8.26, 95% CI (2.23, 30.53), *P* = 0.002, *I*^2^ = 0%, Figure 9), with high subgroup differences observed (hyperkalemia: *P* = 0.003, *I*^2^ = 82.7%; gynecomastia: *P* = 0.003, *I*^2^ = 88.3%, Figure 9).

## 4. Discussion

In this meta-analysis of 15 RCTs involving 11,861 patients, the efficacy and safety of MRA on patients with AMI were evaluated. The principal findings suggest that MRA treatment can improve ventricular remodeling and clinical prognosis in patients with AMI, but the incidence of common adverse events increases.

Post-STEMI patients without LVSD were observed to have statistically significant improvements in cardiac ultrasound parameters. We noted that as treatment duration increased, the extent of reduction in LVEF, LVESVI, and LVEDVI was alleviated or even became nonsignificant. It was evidenced that MRA decreased cardiac aldosterone to suppress collagen synthesis during the acute to subacute phase of AMI [19]. Post-STEMI patients without LVSD potentially reverse early ventricular remodeling and may benefit from MRA. LVEF and E/A ratio are echocardiographic indices to assess left ventricular systolic and diastolic dysfunction [32]. This meta-analysis showed that post-STEMI patients without LVSD treated with MRA had a 2.69% improvement in LVEF, a 15% increase in E/A ratio, a 36% reduction in all-cause mortality, and a 22% reduction in cardiovascular adverse events. Current guidelines strongly recommended the use of MRA in post-AMI patients presenting with HF [33] based on benefits seen in three landmark trials: RALES (Randomized Aldactone Evaluation Study) [34], EPHESUS [11], and EMPHASIS-HF (Eplerenone in Mild Patients Hospitalization And Survival Study in Heart Failure, NCT00232180) [35]. There was limited clinical evidence for MRA used in the treatment of post-STEMI patients without LVSD. MRA are not currently recommended as a standard of care for post-STEMI patients without LVSD. Our findings provide possible evidence for the use of MRA in these patients. The left atrium (LA) is able to pump blood into the left ventricle at end-diastole and help maintain cardiac output, so antiatrial remodeling is essential for AMI patients. MRA treatment showed a little benefit for LA remodeling after AMI [23, 26]. A large number of related studies are needed for further exploration in the future. MRA have shown to affect circulating levels of biomarkers indicating cardiac fibrosis and function such as MMP, PIIINP, and NT-pro BNP [19, 25, 36–40]. Therefore, we call for further investigation on noninvasive indicators in response to MRA to prove its predictive value in cardiac remodeling.

Some studies have shown that early administration of MRA after AMI improves efficacy [13, 28], but the optimal timing of MRA in AMI remains uncertain. We found that the earlier the treatment, the lower the all-cause mortality. Early administration of MRA within 7 days resulted in a 28% reduction in death after randomization. We hypothesize that this is because early application of MRA suppresses deleterious effects resulting from high aldosterone plasma levels early after AMI [7]. These data suggest that there is a window of opportunity in the first days after AMI to maximize the potential beneficial effects of MRA on cardiovascular outcomes.

AMI is divided into STEMI and non-ST-elevation myocardial infarction (NSTEMI). STEMI patients usually have complete coronary obstruction, which is more acute and severe than NSTEMI. Emergency treatment is required to restore patency as soon as possible. For NSTEMI, the artery is usually patent but severely stenosed and does not require urgent reperfusion therapy or aggressive antithrombotic therapy [41]. The ALBATROSS trial [12] found a reduction in death in STEMI patients receiving the rapid MRA regimen, and the REMINDER trial [28] (A Double-Blind, Randomized, Placebo-Controlled Trial Evaluating The Safety And Efficacy Of Early Treatment With Eplerenone In Patients With Acute Myocardial Infarction, NCT01176968) showed that eplerenone used in 1012 low-risk STEMI patients was safe and effective on a composite outcome. Our study showed a 36% reduction in all-cause deaths to provide further support for the use of MRA in STEMI patients. 7990 subjects (NSTEMI 2127; STEMI 5863) from the EPHESUS trial [11] and the ALBATROSS trial [12] showed that NSTEMI patients experienced more all-cause deaths (16.41% vs. 10.90%) under MRA treatment than STEMI patients, and whether MRA was applicable to NSTEMI patients required further investigation.

The present study showed that hyperkalemia was higher in AMI patients treated with MRA (4.71%) than in controls (2.76%). The two longest follow-up trials [11, 25] had similar rates of severe hyperkalemia over 24 and 16 months, with increases of 2.0% and 1.6% over controls, respectively. Hyperkalemia is the most common side effects of MRA, often with arrhythmia as the first manifestation. Therefore, we call on clinicians to prescribe MRA with caution on the basis of adequate assessment of renal function. Close monitoring of serum potassium, creatinine, and ECG during medication can improve safety. Gynecomastia is the most important side effect requiring discontinuation. Spironolactone is more likely to cause gynecomastia due to its lower selectivity for mineralocorticoid receptors than eplerenone and also binds to androgen and progesterone receptors [42]. Recently, nonsteroidal MRA have been developed, including finerenone and esaxerenone, which are expected to reduce the incidence of above adverse events due to strong and highly selective mineralocorticoid receptor inhibition [43, 44].

Coadministration of MRA and angiotensin converting enzyme inhibitors (ACEI) has been considered relatively contraindicated owing to potential hyperkalemia. However, the RALES pilot study [45] and the subsequent RALES trial [34] showed that spironolactone in combination with ACEI significantly reduced mortality in patients with advanced HF but was also safe. Di Pasquale et al. [21] and their previous pilot trial [20] also showed that canrenoate plus captopril combination therapy after AMI was well tolerated and had better beneficial effects. Partial aldosterone escapes during chronic treatment with ACEI alone [46], so aldosterone blockade, alone or in combination with ACEI, has potentially favorable effects on post-AMI patients.

The reperfusion process itself can further lead to myocardial injury [47]. The MINIMIZE STEMI trial [13] was the first study to assess whether spironolactone administered prior to reperfusion provided a benefit against reperfusion injury, which showed no benefit in reducing MI size but improving left ventricular remodeling in STEMI patients at 3 months. Iqbal et al. [48] had highlighted that eplerenone was effective in patients after AMI whether treated with or without percutaneous coronary intervention (PCI). Due to the limited relevant data collected, we are not able to analyze whether MRA can improve reperfusion injury in AMI patients and then affect clinical prognosis. Further prospective studies are warranted. Ongoing Clear-Synergy trial (NCT03048825), a multicenter, international Synergy stent registry embedded in a 2 × 2 factorial design trial of colchicine versus placebo and spironolactone versus placebo in patients with myocardial infarction undergoing primary PCI has been designed to address this issue.

## 5. Limitations

This study to date is the first comprehensive evaluation of MRA use in AMI patients. We believe that we have identified all existing studies that met our inclusion criteria by meticulous search, hence yielding robust results. However, there are several potential limitations. First, subjects may not represent all patients in clinical practice. Second, differences in follow-up duration and medications may be attributed to unremovable heterogeneity. Lastly, selection bias cannot be completely ruled out by only retrieving English articles and published trials. Therefore, we cannot draw definitive conclusions until the present results are further validated in larger more targeted clinical trials.

## 6. Conclusion

Based on current evidence, MRA treatment reduced all-cause mortality and the composite outcome of ventricular arrhythmia, ischemic events, new or worsening HF, cardiovascular deaths, and cardiovascular hospitalizations in post-STEMI patients without LVSD. In addition, post-STEMI patients without LVSD improved ventricular remodeling and cardiac function by MRA. Early administration of MRA within 7 days after AMI resulted in a greater improvement in all-cause mortality and LVEF. Whether early application of MRA is required in post-STEMI patients without LVSD needs further adequately powered RCTs to warrant. The increase in adverse events requires close monitoring.

## Figures and Tables

**Table 1:** Baseline characteristics of trials included in the meta-analysis.

First author (year)	Study design	ITTA	Duration (month)	Jadad points	Country
Rodríguez (1997) [17]	Randomized, double-blind, placebo	Yes	6	6	Chile
Modena (2001) [18]	Randomized, placebo	Yes	12	5	Italy
Pitt (2003) [11]	Randomized, double-blind, placebo	Yes	16	7	Multiple
Hayashi (2003) [19]	Randomized, nonplacebo	No	1	6	Japan
Di Pasquale (2001) [20]	Randomized, double-blind, placebo	No	6	5	Italy
Di Pasquale (2005) [21]	Randomized, double-blind, placebo	No	6	5	Italy
Uzunhasan (2009) [22]	Randomized, double-blind, placebo	Yes	6	7	Turkey
Kayrak (2010) [23]	Randomized, nonplacebo	No	6	5	Turkey
Weir (2011) [24]	Randomized, double-blind, placebo	No	5.5	7	UK
Kampourides (2012) [25]	Randomized, open-labeled, nonplacebo	No	24	6	Greece
Wu (2013) [26]	Randomized, placebo	No	12	6	China
Vatankulu (2013) [27]	Randomized, nonplacebo	Yes	6	5	Turkey
Montalescot (2014) [28]	Randomized, double-blind, placebo	Yes	10.5	7	Multiple
Beygui (2016) [12]	Randomized, open-labeled, blinded endpoint, nonplacebo	Yes	6	5	Multiple
Bulluck (2019) [13]	Randomized, double-blinded, placebo	Yes	3	7	UK

ITTA: intention to treat analysis.

**Table 2:** Baseline characteristics of patients included in the meta-analysis.

Author (year)	Comparison Drug (mg/d)	Patients Number	Cr (mg/dl) K (mmol/l)	LVEF (%) Killip class	Age Male (female)
					MRA/non-MRA
Rodríguez [17] (1997)	SP (75) vs. P	AMI/47	<2.0 NA	NR NR	58.8 (10.8)/58.6 (9.0)^b^ 18 (5)/22 (2)
Modena [18] (2001)	CAN (50) + ACEI vs. ACEI + P	STEMI, 6 h^a^/46	≤2.5 NA	>40 I-III	59.0 (10.0)/62.0 (13.0) 17 (7)/17 (5)
Pitt [11] (2003)	ST + EP (50) vs.ST + P	AMI, LVSD, (3-14 d)/6632	≤2.5 ≤5.0	≤40 NR	64.0 (11.0)/64.0 (12.0) 2380 (939)/2334 (979)
Hayashi [19] (2003)	SP (25) + ACEI vs. ACEI	STEMI, SR, 24 h/150	≤2.0 ≤5.0	>40 I-II	64.4 (1.4)/62.9 (1.4) 49 (16)/51 (18)
Di Pasquale [20] (2001)	ST + CAN (25) + CAP vs.ST + CAP + P	STEMI, 4 h/187	≤2.0 ≤5.0	>40 I-II	63.6 (15.0)/62.8 (16.0) 62 (32)/61 (32)
Di Pasquale [21] (2005)	ST + CAN (25) + CAP vs.ST + CAP + P	STEMI, 4 h/687	≤2.0 ≤5.0	>40 I-II	62.6 (6.0)/62.8 (5.0) 243 (98)/244 (102)
Uzunhasan [22] (2009)	ST + SP (50) vs.ST + P	STEMI, SR, 6-12 h/82	≤2.5 ≤5.0	>40 I-II	52.0 (10.0)/52.0 (10.0) 32 (9)/29 (11)
Kayrak [23] (2010)	ST + SP (25) vs.ST	STEMI, SR, 12 h/142	≤2.0 ≤5.0	≥40 I-II	55.3 (10.0)/57.2 (11.1) 10 (45)/14 (41)
Weir [24] (2011)	ST + EP (50) vs.ST + P	AMI, LVSD (1-14 d)/100	≤2.5 ≤5.0	<40 I	61.0 (12.0)/56.8 (12.0) 37 (13)/40 (10)
Kampourides [25] (2012)	ST + EP (25) vs. ST	STEMI, 24 h/327	≤2.5 ≤5.0	≥40 I	ND
Wu [26] (2013)	ST + SP (20) vs.ST	STEMI, 24 h/616	≤2.5 ≤5.0	>40 I-III	59.8 (11.7)/59.9 (10.3) 193 (69)/192 (74)
Vatankulu [27] (2013)	ST + SP (12.5-5) vs. ST	STEMI, SR/110	≤2.0 <5.5	≥40 I-II	56.0 (10.1)/57.0 (11.0) 89 (15)/45 (11)
Montalescot [28] (2014)	ST + EP (50) vs. ST + P	STEMI, 24 h/1012	<2.5 NA	>40 NR	58.5 (10.8)/57.8 (11.0) 420 (86)/403 (103)
Beygui [12] (2016)	ST + SP (25) vs. ST	AMI, 72 h/1603	<2.5 <5.5	>40 I-IV	58.0 (13.0)/58.0 (13.0) 673 (129)/658 (143)
Bulluck [13] (2019)	SP (50) vs. P	STEMI, 12 h/70	NA <5.0	>40 I	62.0 (10.0)/60.0 (13.0) 33 (5)/27 (5)

^a^Time from disease onset to trial entry; ^b^mean (standard deviation). EP: eplerenone; SP: spironolactone; CAN: canrenoate; CAP: captopril; ST: standard therapy; ACEI: angiotensin converting enzyme inhibitors; P: placebo; LVEF: left ventricular ejection fraction; LVSD: left ventricular systolic dysfunction; AMI: acute myocardial infarction; STEMI: ST-segment elevation myocardial infarction; MRA: mineralocorticoid receptor antagonists; SR: successful reperfusion; ND: not defined; NR: not restricted; NA: not available; Cr: creatinine; K: kalium.

**Table 3:** Other statistical results of the meta-analysis for comparison between the two groups.

						Heterogeneity	
Outcomes	Trials	*N*	RR/MD	95% CI	*P* value	*I* ^2^ (%)	*P* value
Deaths due to HF	5	9259	0.77	(0.61, 0.97)	0.03	0	0.80
Serum potassium level (mmol/l)	9	2949	0.14	(0.06, 0.23)	0.001	93	<0.001
Serum creatinine level (mg/dl)	7	2733	0.02	(0.00, 0.04)	0.02	69	0.004
Hypokalemia	3	7702	0.42	(0.19, 0.95)	0.04	64	0.06
LVEF (%)	7	1754	2.69	(2.44, 2.93)	<0.001	36	0.15
LVEDVI (ml/m^2^)	6	1246	-3.19	(-4.97, -1.41)	<0.001	0	0.49
LVESVI (ml/m^2^)	6	1270	-4.52	(-8.21, -0.83)	0.02	96	<0.001
LVEDD (cm)	4	854	-0.11	(-0.22, 0.00)	0.05	60	0.06
LVESD (cm)	4	854	-0.15	(-0.43, 0.14)	0.31	93	<0.001
E/A ratio	4	1200	0.15	(0.10, 0.19)	<0.001	55	0.06
EDT (m/s)	4	1200	6.25	(3.25, 9.26)	<0.001	0	0.46

*N*: number; MD: mean difference; RR: relative ratio; CI: confidence interval; *I*^2^: inconsistency index; HF: heart failure; LVEF: left ventricular ejection fraction; LVEDVI: left ventricular end-diastolic volume index; LVESVI: left ventricular end-systolic volume index; LVEDD: left ventricular end-diastolic diameter; LVESD: left ventricular end-systolic diameter; E/A: mitral diastolic early flow velocity E to mitral late flow velocity A; EDT: E-wave deceleration time.

## Data Availability

All data during the course of this meta-analysis were included in the article.

## Supplementary Materials

Supplementary Materials The PRISMA 2020 Checklist. The full details of search strategy in the meta-analysis. Kappa statistic between two independent reviewers of this meta-analysis.
